# Economic Analysis of Infectious Disease Consultation for *Staphylococcus aureus* Bacteremia Among Hospitalized Patients

**DOI:** 10.1001/jamanetworkopen.2022.34186

**Published:** 2022-09-29

**Authors:** Elina Eleftheria Pliakos, Panayiotis D. Ziakas, Eleftherios Mylonakis

**Affiliations:** 1 Infectious Diseases Division, Warren Alpert Medical School of Brown University, Rhode Island Hospital, Providence, Rhode Island; 2 Division of General Internal Medicine, Perelman School of Medicine at the University of Pennsylvania, Philadelphia

## Abstract

**Importance:**

*Staphylococcus aureus* bacteremia is associated with a significant burden of mortality, morbidity, and health care costs. Infectious disease consultation may be associated with reduced mortality and bacteremia recurrence rates.

**Objective:**

To evaluate the cost-effectiveness of infectious disease consultation for *Staphylococcus aureus* bacteremia.

**Design, Setting, and Participants:**

In this economic evaluation, a decision-analytic model was constructed comparing infectious disease consult with no consult*.* The population was adult hospital inpatients with *Staphylococcus aureus* bacteremia diagnosed with at least 1 positive blood culture. Cost-effectiveness was calculated as deaths averted and incremental cost-effectiveness ratios. Uncertainty was addressed by plotting cost-effectiveness planes and acceptability curves for various willingness-to-pay thresholds. Costs and outcomes were calculated for a time horizon of 6 months. The analysis was performed from a societal perspective and included studies that had been published by January 2022.

**Interventions:**

Patients received or did not receive formal bedside consultation after positive blood cultures for *Staphylococcus aureus* bacteremia.

**Main Outcomes and Measures:**

The main outcomes were incremental difference in effectiveness (survival probabilities), incremental difference in cost (US dollars) and incremental cost-effectiveness ratios (US dollars/deaths averted).

**Results:**

This model included 1708 patients who received consultation and 1273 patients who did not. In the base-case analysis, the cost associated with the infectious disease consult strategy was $54 137.4 and the associated probability of survival was 0.77. For the no consult strategy, the cost was $57 051.2, and the probability of survival was 0.72. The incremental difference in cost between strategies was $2913.8, and the incremental difference in effectiveness was 0.05. Overall, consultation was associated with estimated savings of $55 613.4/death averted (incremental cost-effectiveness ratio, −$55613.4/death averted). In the probabilistic analysis, at a willingness-to-pay threshold of $50 000, infectious disease consult was cost-effective compared with no consult in 54% of 10 000 simulations. In cost-effectiveness acceptability curves, the consult strategy was cost-effective in 58% to 73%) of simulations compared with no consult for a willingness-to-pay threshold ranging from $0 to $150 000.

**Conclusions and Relevance:**

These findings suggest that infectious disease consultation may be a cost-effective strategy for management of *Staphylococcus aureus* bacteremia and that it is associated with health care cost-savings.

## Key Points

**Question:**

Is infectious disease consultation cost-effective for the management of *Staphylococcus aureus *bacteremia*?*

**Findings:**

In this economic evaluation, infectious disease consultation was associated with estimated savings of $55 613.4/death averted and was cost-effective in 54% of 10 000 simulations for a willingness-to-pay of threshold $50 000 compared with no infectious disease consultation.

**Meaning:**

These findings suggest that the use of infectious disease consultation for the management of *Staphylococcus aureus* bacteremia may be a cost-effective strategy associated with significant health care cost savings.

## Teaser

This economic evaluation investigates the savings associated with infectious disease consultation for management of *Staphylococcus aureus *bacteremia among hospitalized patients.

## Introduction

*Staphylococcus aureus* bacteremia (SAB) is associated with a significant burden of mortality, morbidity, and health care costs.^1,2,3^ The Centers for Disease Control and Prevention estimated the in-hospital mortality of SAB at 18%.^4^ Moreover, SAB is associated with increased rates of recurrence, hospital length of stay, and total hospital charges.^5,6^ Optimizing the management of SAB is essential to improve patient outcomes and curb health care expenditures.

Infectious disease (ID) consultation for SAB was associated with more appropriate antimicrobial therapy and diagnostic tests, such as follow-up blood cultures and echocardiography,^7^ as well as reduced mortality and bacteremia recurrence rates.^8,9,10,11,12,13,14,15,16^ Evidence-based clinical management by an ID specialist allows for early source control, which is associated with reduced complications and recurrence^8,9,10,11,12,13,14,15,16,17^ and potentially lower health care expenditures. Given the current emphasis among clinical decision-makers on high-value affordable care^18,19,20^ that aims to optimize allocation of resources while improving patient outcomes, the aim of this study was to perform a cost-effectiveness analysis comparing use of ID consultation with no ID consultation for the management of SAB.

## Methods

As per established Rhode Island Hospital policies, studies based on public data in the literature do not require institutional review board approval or informed consent. This economic evaluation followed the recommendations made by the Consolidated Health Economic Evaluation Reporting Standards (CHEERS) reporting guideline^21^ and guidelines reported in 2017 by the Second Panel on Cost-Effectiveness Analysis.^22^

### Model Structure

**Figure 1.**
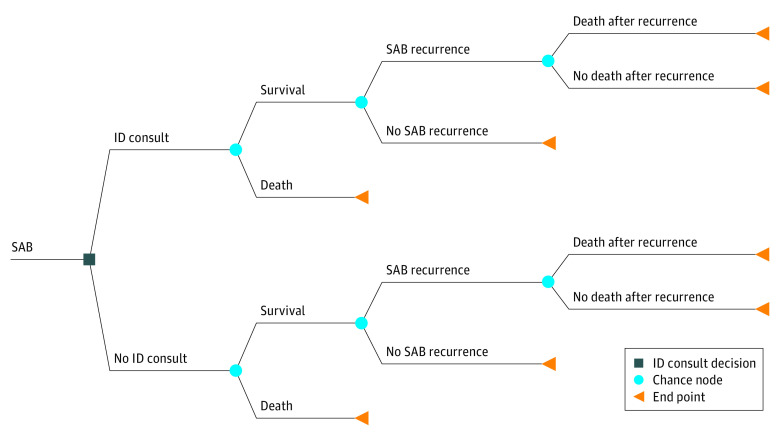
Decision Tree Model ID indicates infectious disease; SAB, *Staphylococcus aureus* bacteremia.

We constructed a decision-analytic model (Figure 1) assessing the cost-effectiveness of an ID consult compared with no ID consult for the management of SAB. ID consult consisted of formal bedside consultation by an ID specialist. The patient population of our analysis consisted of adult hospital inpatients with SAB diagnosed as having at least 1 positive blood culture. Costs and outcomes were calculated for a time horizon of 6 months, and the analysis was performed from a societal perspective.

Our study included an outcome inventory, as recommended by guidelines.^22^ The outcome inventory is a checklist of health and nonhealth outcomes that were considered in this analysis, and it can be found in Table 1.^8,9,10,11,12,13,14,15,16,23,24,25,26,27,28,29,30,31^ Cost data were obtained from sources that reported values in US dollars. Mortality included 90-day mortality, and readmission was defined as recurrence within 90 days. Most North American and European studies that were available in the literature used these time variables, so we used them as well to minimize reporting variability between studies. The model was developed using TreeAge Pro version 2019 R2.0 software (TreeAge). This analysis included studies that had been published up to January 2022.

**Table 1.** Outcome Inventory

Type of outcome	Included in this analysis from the societal perspective?
Formal health care sector: health outcomes	
	
Mortality	Yes
Medical costs	
Paid by third party	Yes
Paid by patients out of pocket	Yes
Future related medical costs (payers and patients)	Yes
Future unrelated medical costs (payers and patients)	No
Non–health care sector: productivity	
Labor market earnings lost due to absence from work	Yes
Uncompensated household production, patient	No

### Model Inputs: Assigning Probabilities

To identify studies that provided data on the effectiveness associated with ID consult compared with no ID consult for SAB, we used the most recently published and relevant systematic reviews and meta-analyses.^7,32,33^ A meta-analysis by Vogel et al^7^ was used as the basis of our analysis. Using search terms in this study, we expanded the search to January 2022. Characteristics of studies that were used to obtain effectiveness^8,9,10,11,12,13,14,15,16^ can be found in the eTable in the Supplement. We included studies on SAB that compared bedside ID consultation with no consultation and provided data on 90-day mortality. For length of hospital stay, we used a study by Bai et al^8^ given that it was the most recent study conducted in North America with the largest population size. For the 90-day probability of mortality after recurrence, we used a study by Szubert et al.^23^ Probability estimates and CIs for mortality,^8,9,10,11,12,13,14,15,16^ recurrence,^9,11,13,14,15,16^ echocardiography,^8,9,10,11,12,13,15,16^ and follow-up blood cultures^8,11,13,16^ were obtained by pooling with the use of random-effects meta-analysis (DerSimonian^34^) using MedCalc software version 19.8 (MedCalc Software). The 95% CIs correspond to the ranges presented in Table 2. This method was chosen because it accounts for the considerable interstudy differences and heterogeneity among the included studies.^34^

**Table 2.** Model Inputs and Baseline Estimates

	Base case value (range)^a^	Distribution (SD)^b^	Source
**Probability**			
Mortality with ID consult	0.22 (0.15-0.30)	0.22 (0.03)	Bai et al,^8^ 2015; Forsblom et al,^9^ 2013; Honda et al,^10^ 2010; Jenkins et al,^11^ 2008; Jogenfors et al,^12^ 2014; Lopez-Cortes et al,^13^ 2013; Pragman et al,^14^ 2012; Rieg et al,^15^ 2009; Saunderson et al,^16^ 2015
Mortality without ID consult	0.27 (0.21-0.34)	0.27 (0.02)	Bai et al,^8^ 2015; Forsblom et al,^9^ 2013; Honda et al,^10^ 2010; Jenkins et al,^11^ 2008; Jogenfors et al,^12^ 2014; Lopez-Cortes et al,^13^ 2013; Pragman et al,^14^ 2012; Rieg et al,^15^ 2009; Saunderson et al,^16^ 2015
Bacteremia recurrence with ID consult	0.04 (0.02-0.07)	0.04 (0.01)	Forsblom et al,^9^ 2013; Jenkins et al,^11^ 2008; Lopez-Cortes et al,^13^ 2013; Pragman et al,^14^ 2012; Rieg et al,^15^ 2009; Saunderson et al,^16^ 2015
Bacteremia recurrence without ID consult	0.06 (0.03-0.10)	0.06 (0.01)	Forsblom et al,^9^ 2013; Jenkins et al,^11^ 2008; Lopez-Cortes et al,^13^ 2013; Pragman et al,^14^ 2012; Rieg et al,^15^ 2009; Saunderson et al,^16^ 2015
90-d Mortality after SAB recurrence	0.19 (0.10-0.38)	0.19 (0.05)	Szubert et al,^23^ 2019
Follow-up cultures with ID consult	0.77 (0.47-0.97)	0.77 (0.08)	Bai et al,^8^ 2015; Jenkins et al,^11^ 2008; Lopez-Cortes et al,^13^ 2013; Saunderson et al,^16^ 2015
Follow-up cultures with no ID consult	0.57 (0.38-0.75)	0.57 (0.06)	Bai et al,^8^ 2015; Jenkins et al,^11^ 2008; Lopez-Cortes et al,^13^ 2013; Saunderson et al,^16^ 2015
Echocardiography with ID consult	0.73 (0.62-0.82)	0.73 (0.03)	Bai et al,^8^ 2015; Forsblom et al,^9^ 2013; Honda et al,^10^ 2010; Jenkins et al,^11^ 2008; Jogenfors et al,^12^ 2014; Lopez-Cortes et al,^13^ 2013; Rieg et al,^15^ 2009; Saunderson et al,^16^ 2015
Echocardiography with no ID consult	0.39 (0.25-0.54)	0.39 (0.05)	Bai et al,^8^ 2015; Forsblom et al,^9^ 2013; Honda et al,^10^ 2010; Jenkins et al,^11^ 2008; Jogenfors et al,^12^ 2014; Lopez-Cortes et al,^13^ 2013; Rieg et al,^15^ 2009; Saunderson et al,^16^ 2015
Catheter-related bacteremia with ID consult	0.05 (0.03-0.10)	0.05 (0.01)	Jenkins et al,^11^ 2008
Catheter-related bacteremia without ID consult	0.11 (0.06-0.22)	0.11 (0.03)	Jenkins et al,^11^ 2008
Lab toxicity with ID consult	0.09 (0.05-0.18)	0.09 (0.02)	Jenkins et al,^11^ 2008
Lab toxicity without ID consult	0.16 (0.08-0.32)	β (0.16; 0.04)	Jenkins et al,^11^ 2008
**Costs, $**			
SAB recurrence	20 971.8 (10 485.9-41 943.6)	20 971.8 (5242.95)	Inagaki et al,^5^ 2019
ID consult	278.5 (139.3-557.0)	278.5 (69.6)	Stephens et al,^24^ 2013
			Tseng et al,^31^ 2018
Antimicrobial treatment	584.8 (292.4-1169.6)	584.8 (190.0)	Stephens et al,^24^ 2013
Echocardiography for SAB	857.7 (428.9-1715.4)	857.7 (214.4)	Rosen et al,^26^ 1999
Blood culture	40.2 (20.1-80.4)	40.2 (10.1)	Skoglund et al,^27^ 2019
Hospitalization/d for Rhode Island	3099.5 (1549.8-6199.0)	3099.5 (774.9)	Foundation KF,^25^ 2022
Catheter-related infection	16 506.2 (8253.1-33 012.4)	16 506.2 (4126.6)	Warren et al,^28^ 2006
Lab toxicity	2839.2 (1419.6-5678.4)	2839.2 (709.8)	Patel et al,^29^ 2014
Lost productivity/d	144.3 (72.2-288.6)	144.3 (36.1)	Statistics BoL,^30^ 2021
**LOS, d**			
With ID consult	16.0 (9.0-33.0)	16.0 (4.0)	Bai et al,^8^ 2015
Without ID consult	17.0 (9.0-36.0)	17.0 (4.5)	Bai et al,^8^ 2015

Abbreviations: ID, infectious disease; LOS, length of stay; SAB, *Staphylococcus aureus* bacteremia.

^a^ Ranges for probabilities were obtained from pooling via meta-analysis and correspond to 95% CIs.

^b^ Distributions are β for probabilities and γ for LOS and costs.

### Model Inputs: Assigning Costs

Costs were obtained from the literature and were adjusted to January 2022 US dollars using the Consumer Price Index Inflation calculator provided by the Bureau of Labor Statistics.^35^ The ID specialist cost for an inpatient visit was estimated at $278.51.^24^ The cost of hospitalization was estimated by multiplying the cost of hospitalization/day for the state of Rhode Island ($3099.5), provided by the Kaiser Family Foundation,^25^ by the length of hospital stay for patients with an ID consult (16 days) or without an ID consult (17 days).^8,11,16^

The cost for bacteremia recurrence was estimated at $20971.8. This value was obtained from a study by Inagaki et al^5^ that used methicillin-sensitive *S. aureus* (MSSA) and methicillin-resistant *S. aureus* (MRSA) data from the Nationwide Readmissions Database. The cost of echocardiography for SAB was estimated to be $857.7,^26^ while the cost of a blood culture was estimated at $40.2.^27^ We assumed that each patient would receive 3 follow-up blood cultures.^36,37^ Inpatient antimicrobial drug costs, including drug treatment and drug administration costs, were estimated at $584.8 based on a study by Stephens et al.^24^ To model complications, we used data for catheter-related bacteremia and lab toxicity obtained by Jenkins et al.^11^ The cost of catheter-related bacteremia was estimated at $16 506.2,^28^ while the cost of toxicity was estimated at $2839.2.^29^ The cost of lost productivity per day was estimated by multiplying the usual daily earning ($144.3) for US salary workers, provided from the US Department of Labor,^30^ by the length of stay associated with SAB with ID consult or SAB without ID consult.^8,11,16^

### Outcome

In the base case analysis, our primary outcome was the incremental cost-effectiveness ratio (ICER), defined as the ratio of the incremental cost between strategies (ID consult vs no ID consult) over their incremental difference in effectiveness.^21^ The incremental cost was defined as the excess cost of ID consult for SAB compared with the cost of no ID consult. In turn, the incremental effectiveness was defined in terms of deaths averted.

### Statistical Analysis

The robustness of our model was evaluated with the use of deterministic (1-way sensitivity) and probabilistic sensitivity analysis (Monte Carlo). In the 1-way sensitivity analysis,^38^ each parameter was tested across a range of point estimates, while in the probabilistic analysis, we varied all parameters of the model simultaneously. The base-case estimates, ranges, and distributions for parameters are presented in Table 2.

Probabilities were modeled as β distributions and uniform distributions, while costs and length of stay were modeled as γ distributions, as recommended by guidelines.^39^ When a range was not available for a variable, we approximated it by allowing the variable to vary between 50% and 200% of its base case value.^40^ If an SD was not available, it was estimated by dividing the range by 6, as suggested for data that do not follow the normal distribution (approximation obtained with the use of Chebyshev inequality).^41^

In the Monte Carlo analysis,^42^ the model was run 10 000 times,^40^ and each time a value from the predetermined distributions (Table 2) was randomly selected for each variable. For example, the base case value (range) and β distribution (SD) for mortality probability were 0.22 (0.15-0.30) and 0.22 (0.03) with ID consult and 0.27 (0.21-0.34) and 0.27 (0.02) without ID consult, while the base case value (range) and γ distribution (SD) for hospital length of stay were 16.0 (9.0-33.0) days and 16.0 (4.0) days with ID consult and 17.0 (9.0-36.0) days and 17.0 (4.5) days without ID consult (Table 2). The results of each simulation were plotted on an incremental cost-effectiveness plane as points with coordinates (x, y), with x representing incremental effectiveness and y representing incremental cost. Points located within the southeast quadrant of the graph were considered to be cost-effective and dominant.^43^ Finally, cost-effectiveness acceptability curves were used to evaluate the cost-effectiveness for various willingness-to-pay thresholds.^44^ These are graphs summarizing the association of uncertainty with the result of our economic evaluation by plotting a range of willingness-to-pay thresholds on the x-axis ($0-$150 000) against the probability that ID consult strategy would be cost-effective in the y-axis.

## Results

This model included data from 1708 patients who received ID consultation and 1273 patients who did not. Using our evidence-based approach, the base-case analysis for the cost for the ID consult strategy was calculated as $54 137.4, while the probability of survival was estimated to be 0.77. The ID consult strategy included the costs of ID specialist,^24,31^ antimicrobial treatment,^24^ echocardiography,^26^ repeat blood cultures,^27^ hospitalization,^25^ SAB recurrence,^5^ and lost productivity,^30^ while the probability of survival was estimated by pooling available studies.^8,9,10,11,12,13,14,15,16^ Similarly, for the no ID consult strategy, the cost was calculated to be $57 051.2, and the probability of survival was 0.72.

The incremental difference in cost between strategies was $2913.8, and the incremental difference in effectiveness was 0.05. ID consult was associated with 1 fewer death per 20 patients treated and a savings of $55 613.4/death averted (ICER, −$55 613.4/death averted) compared with no ID consult, suggesting that it was the more cost-effective strategy.

**Figure 2.**
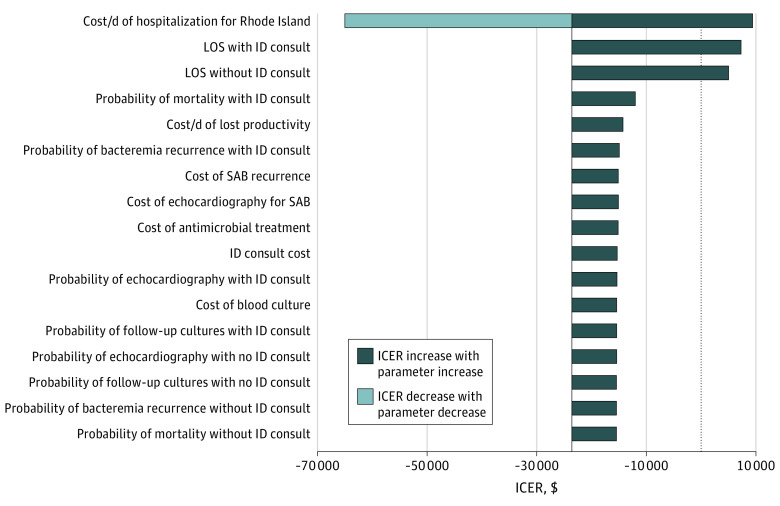
Sensitivity Analysis This tornado diagram is a summary of the 1-way sensitivity analysis. From top to bottom, it presents variables associated with the greatest change in the incremental cost-effectiveness ratios (ICERs). ID indicates infectious disease; LOS, length of stay.

The sensitivity analysis, which allowed us to test each model variable for thresholds by varying each base-case value within the limits specified in Table 2, suggested that in clinical practice, the no ID consult would become the cost-effective strategy only in rare cases when the probability of death with no ID consult was less than 0.22. In addition, it suggested that no ID consult would become the cost-effective strategy only if the length of stay with ID consult was longer than 16.9 days, the length of stay without ID consult was shorter than 16.1 days, or the probability of death with ID consult was greater than 0.27. The findings of the sensitivity analysis are summarized in the tornado diagram (Figure 2), which is a graphical representation of how variations in each model variable were associated with the cost-effectiveness output.

In the probabilistic analysis, the mean cost for the strategy that included ID consult was estimated to be $55 273.3 (95% CI, $54 916.0-$55 630.6) and the mean cost for the strategy that included no ID consult was estimated to be $59 328.4 (95% CI, $58 937.6-$59 719.1). In addition, the cost-effectiveness plane (eFigure in the Supplement), which aimed to show the uncertainty around the cost-effectiveness outcomes, ID consult was located in the dominant and cost-effective quadrants in 54% of 10 000 simulations.

**Figure 3.**
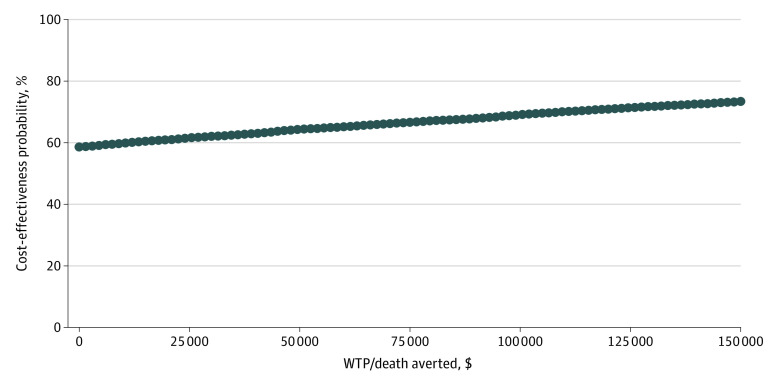
Cost-effectiveness Acceptability by Willingness-to-Pay (WTP) Threshold This curve shows the probability that infectious disease consult was a cost-effective strategy compared with no infectious disease consult, the baseline strategy, for a range of WTP thresholds.

The cost-effectiveness acceptability curve (Figure 3) shows the probability that ID consult was cost-effective compared with no ID consult for various willingness-to-pay thresholds. ID consult was cost-effective in 58% of simulations for a willingness-to-pay threshold of $0 and in 73% of simulations for a willingness-to-pay threshold of $150 000.

## Discussion

Economic decision models that incorporate and simulate information about the effectiveness and costs associated with different strategies can help inform medical decision-making.^22^ SAB is associated with significant health care costs.^5,6^ is difficult to elucidate whether ID consult is a cost-effective strategy for SAB without economic modeling due to the plethora of factors that may be associated with inpatient and postdischarge costs, such as length of stay, additional testing, complications, and recurrence episodes. Our economic evaluation study suggests that ID consultation may be a cost-effective strategy for the management of SAB given that it was associated with 1 fewer death per 20 patients treated and an estimated savings of $55 613.4/death averted (ICER, −$55 613.4/death averted).

There is a paucity of studies in the literature about the value of ID specialist interventions for patient care, possibly due to challenging study design and the high cost of undertaking these investigations.^45^ Nevertheless, our cost findings are in line with previous analyses that suggest the economic benefit associated with ID consultation. Of note, a study by Schmitt et al^45^ that used administrative Medicare claims data and propensity score matching estimated that ID specialty intervention was associated with lower health care costs for various types of infection, including SAB. The benefit was greatest when the ID specialist was involved within the first 2 days of hospital admission.^45^ On a similar note, a previous study,^37^ we found that antimicrobial stewardship programs were associated with increased benefits of diagnostic testing for bloodstream infections, further suggesting the benefit of expert opinion in the management of bacteremia. In a previous decision analysis study by Lundberg et al^46^ that investigated a different question for SAB (the cost-effectiveness of 4-week treatment for SAB compared with 2-week treatment), the excess cost per life-year saved was $18 000 (1998 value; adjusted value for 2022, $31 315.99).

The increased effectiveness associated with the ID consult strategy may be explained by the association of ID specialist involvement with better adherence to best practices and improved patient outcomes in SAB.^7,32^ ID specialists may mitigate some factors associated with poor prognosis of SAB, such as inadequate or inappropriate antibiotic treatment and an undetermined focus of infection or nonremoval of an infectious focus.^32,47^ Specifically, timing and choice of antibiotic have been shown to be important because delay in treatment or inappropriate choice of antimicrobial were associated with increased mortality.^47^ Similarly, noneradicable or noneradicated foci have been found to be independent factors associated with mortality.^47^ ID physicians are more likely than internists to order follow-up blood cultures, echocardiography studies, and appropriate antibiotic therapy and to undertake source identification and control.^7,32^ Moreover, a 2020 study^48^ found that daily ID consultation was associated with more comprehensive treatment of patients compared with weekly consultation and globally decreased antibiotic consumption.

Results of our sensitivity analysis suggest that length of hospital stay was associated with the outcomes of our analysis. The decreased cost associated with the ID consult strategy may be explained by the association of ID consult with decreased hospital length of stay, even though it was also associated with increased costs, such as those for the physician, follow-up blood cultures, and echocardiography.

### Limitations

This study has several limitations, so there are certain considerations to be taken into account when applying the results of our study. For patients who die early in their hospital stay, ID consultation may not be possible. Similarly, ID consultation may not be possible in settings with limited resources, and future studies should investigate if telemedicine could mitigate this issue. Additionally, there may be differences in the training and practices of ID specialists in different regions. This is supported by a survey by the Infectious Diseases Society of America, which suggested that although most ID physicians performed repeat blood cultures and echocardiography and treated patients with intravenous therapy for at least 14 days, there was still considerable variation in practices by years of experience, geographic region, and practice environment.^49^

There are also limitations that should be considered given that our study is a decision model. Due to lack of available data, we were unable to distinguish between bacteremia due to MSSA and bacteremia due to MRSA. Moreover, although we modeled complications, we did not have enough data to perform a thorough analysis of adverse events. Similarly, we did not have information to simulate ICU admission. Our model additionally does not include potential benefits associated with curbside ID consultations that may have stealthily been associated with outcomes in the no ID consult group.^50^ In addition, we have accounted for potential variations in base-case model inputs with sensitivity and probabilistic analyses. Furthermore, as recommended by guidelines,^22^ we conducted this study from the perspective of society, which includes hospital costs and societal costs. We included an outcome inventory and lost productivity costs, but we have not accounted for losses associated with uncompensated household production that may be associated with further increase in the cost-effectiveness of the ID consult strategy. Moreover, although we used effectiveness data from outside the US, the cost values used were US specific, and our cost-effectiveness findings should be applied to other countries with caution and appropriate adjustments. Similarly, most data were from large academic settings and need to be validated in other settings, including smaller community hospitals with limited resources.

## Conclusions

Given that SAB is associated with high morbidity and mortality, it is important to develop strategies for cost-effective diagnosis and treatment. By combining data from multiple studies and developing a decision-analytic model, this economic evaluation study found that ID consultation was a cost-effective strategy for the management of SAB. This finding involved the survival benefit, lower recurrence, and lower length of stay associated with involving ID specialists in SAB care. Moreover, ID consultation may be associated with positive long-term outcomes, such as reduced antimicrobial resistance with the appropriate choice of antimicrobial agents that we did not account for in this model. In the future, a randomized clinical trial conducted in conjunction with a cost-effectiveness analysis could provide highly controlled data on the economic benefits associated with ID consultation. In addition, future studies could evaluate differences between MSSA and MRSA and the potential additional benefits associated with antimicrobial stewardship programs. These studies could also have longer follow-up periods to evaluate the long-term cost and health outcomes associated with SAB. In addition, they could assess the benefits associated with ID consultation in the treatment of outpatient SAB.

## Notes

Supplement. **eTable.** Characteristics of Studies Used to Obtain Base-Case Effectiveness Estimates **eFigure.** Incremental Cost-effectiveness Plane for Infectious Disease Consult vs No Consult
