# Supplementary material for: Economic Analysis of Infectious Disease Consultation for Staphylococcus aureus Bacteremia Among Hospitalized Patients
Source: JAMA Netw Open. 2022 Sep 29;5(9):e2234186. doi: 10.1001/jamanetworkopen.2022.34186 (PMC9523499; doi:10.1001/jamanetworkopen.2022.34186)
Supplement: Supplement. — eTable. Characteristics of Studies Used to Obtain Base-Case Effectiveness Estimates eFigure. Incremental Cost-effectiveness Plane for Infectious Disease Consult vs No Consult [file jamanetwopen-e2234186-s001.pdf]

Supplemental Online Content

Pliakos EE, Ziakas PD, Mylonakis E. Economic analysis of infectious disease consultation for *Staphylococcus aureus* bacteremia among hospitalized patients. *JAMA Netw Open*. 2022;5(9):e2234186. doi:10.1001/jamanetworkopen.2022.34186

eTable. Characteristics of Studies Used to Obtain Base-Case Effectiveness Estimates

eFigure. Incremental Cost-effectiveness Plane for Infectious Disease Consult vs No Consult

This supplemental material has been provided by the authors to give readers additional information about their work.

© 2022 Pliakos EE et al. *JAMA Network Open*.

eTable. Characteristics of Studies Used to Obtain Base-Case Effectiveness Estimates

Study Author	Study Design, Duration of Study, Country	Mean or Median age (in years)	Number of participants with male gender	Number of participants with MRSA	ID consult Total Population	No ID consult total population	Timing of ID consultation
Bai 2015	Retrospective, cohort, multicenter, 36 months, Canada	ID consult: 63<br>No ID consult: 68	ID consult: 332<br>No ID consult: 213	145	506	341	Within 7 days of blood culture collection or ID specialist as the responsible physician
Forsblom 2013	Retrospective, cohort, single-center, 60 months, Finland	ID consult: 53.2<br>No ID consult: 55.2	ID consult: 160<br>No ID consult: 21	0	244	26	Within 1 week after the first positive blood culture
Honda 2010	Retrospective, case-control, single-center, 36 months, USA	ID consult: 56<br>No ID consult: 56	ID consult: 56<br>No ID consult: 133	185	111	230	78% were obtained within 5 days after the first positive blood culture
Jenkins 2008	Prospective, case-control, single center, 2x12 months, USA	ID consult: 47<br>No ID consult: 50	ID consult: 87<br>No ID consult: 61	78	100	134	Typically within 48 h of collection of the initial positive culture specimen
Jogenfors 2013	Retrospective, cohort, single-center, Sweden	ID consult: -<br>No ID consult: -	ID consult: 62<br>No ID consult: 35	0	100	58	From first positive blood culture
Lopez-Cortes 2013	Prospective, quasi-experimental, multicenter, 2x6 months, Spain	ID consult: 67<br>No ID consult: 66	ID consult: 140<br>No ID consult: 198	102	162	287	From first positive blood culture
Pragmann 2012	Retrospective, cohort, single-center, 77 months, USA	ID consult: 66<br>No ID consult: 68	ID consult: 178<br>No ID consult: 52	136	149	36	Not specified
Rieg 2009	Retrospective, cohort, single-center, 72 months, Germany	ID consult: 61%>60<br>No ID consult: 61%>60	ID consult: -<br>No ID consult: -	67	300	131	Primary physician was alerted of positive blood cultures within 36h and could call ID consult at their discretion
Saunderson 2015	Observational, cohort, single center, 78 months, UK	ID consult: 64.8<br>No ID consult: 65.8	ID consult: 103<br>No ID consult: 184	127	36	30	Initial consult at the diagnosis of SAB

© 2022 Pliakos EE et al. *JAMA Network Open*.

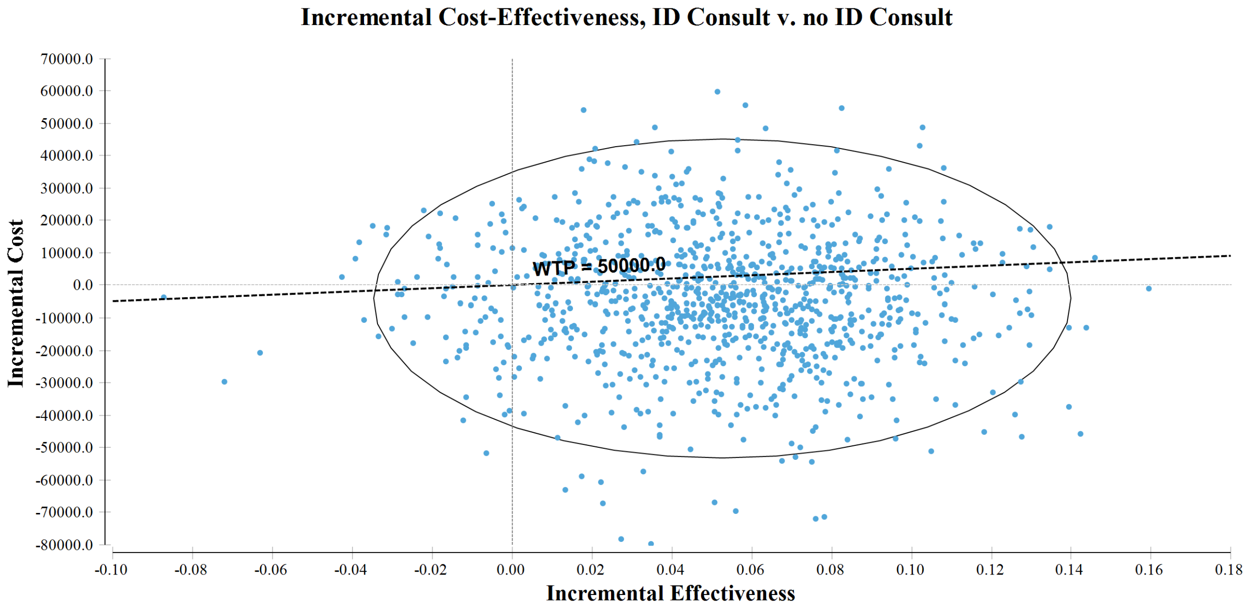

eFigure 1. Incremental Cost-effectiveness Plane for Infectious Disease Consult vs No Consult

The y-axis represents incremental cost while the x-axis represents incremental effectiveness.

© 2022 Pliakos EE et al. *JAMA Network Open*.
